# Evaluating Postural Instability in Patients With Persistent Postural-Perceptual Dizziness (PPPD) Exposed to Moving Visual Stimuli

**DOI:** 10.7759/cureus.89886

**Published:** 2025-08-12

**Authors:** Fumiyuki Goto, Koichiro Wasano, Kanako Masuda, Yoshiharu Yamanobe, Kohei Mizuno, Riichi Uoshima, Chihiro Yagi, Arata Horii

**Affiliations:** 1 Otolaryngology - Head and Neck Surgery, Tokai University School of Medicine, Isehara, JPN; 2 Otolaryngology - Head and Neck Surgery, Tokai University, Isehara, JPN; 3 Otolaryngology - Head and Neck Surgery, National Hospital Organization Tokyo Medical Center, Meguro, JPN; 4 Otolaryngology - Head and Neck Surgery, Niigata University Hospital, Niigata, JPN

**Keywords:** persistent postural-perceptual dizziness (pppd), postural instability, posturography, visually-induced dizziness, visual stimuli

## Abstract

Introduction

This study investigated postural instability in patients with persistent postural-perceptual dizziness (PPPD) in response to moving visual stimuli, using posturography to assess visual-ratio-derived sway responses. While visual factors exacerbate symptoms, the neural mechanisms remain unclear.

Methods

We studied six individuals with PPPD, six healthy controls (HCs), and 35 patients with various vestibular dysfunctions lasting more than three months. Using posturography, we measured sway area with eyes open and closed for 60 seconds. Participants fixated on visual stimuli for 60 secs: a checkerboard, vertical stripes, or optic flow. Measurements were taken during stimulation, calculating visual ratios (VRs) with open eyes.

Results

No significant pre- or post-stimuli differences in sway area were found. However, VR was significantly higher in PPPD patients compared to other disease groups, indicating increased instability during visual stimuli, consistent with visually induced dizziness.

Conclusion

Postural instability during moving visual stimuli suggests a potential objective diagnostic tool for PPPD. These findings offer insights into the neural mechanisms of PPPD symptom exacerbation by visual stimuli and support the utility of visual-stimulus-based posturography as an adjunctive diagnostic tool for PPPD.

## Introduction

The 11th revision of the International Classiﬁcation of Diseases describes persistent postural-perceptual dizziness (PPPD) as a common chronic vestibular syndrome characterized by dizziness, unsteadiness, and non-spinning vertigo persisting for longer than 90 days that is typically preceded by acute vestibular disorders [1]. The symptoms of PPPD are aggravated by an upright posture or walking, active or passive movement, and exposure to moving or visually complex stimuli.

Since PPPD was only recently defined, its prevalence currently remains unknown. The objective diagnosis remains difficult because no standardized biomarkers or quantitative tests are currently validated for PPPD. However, it is considered to affect a large number of patients with chronic balance disorders. Its symptoms may be debilitating and have a profound impact on quality of life.

Difficulties are associated with objectively diagnosing PPPD. Previous studies defined the characteristics of PPPD using clinical examinations [2-4]. Yagi et al. [2] investigated its characteristics using the head roll-tilt subjective visual vertical (HT-SVV) test. They examined 61 patients with PPPD, 10 with unilateral vestibular hypofunction (UVH), and 11 with psychogenic dizziness (PD) who had vestibular symptoms for more than three months. While conventional vestibular tests, including upright position (UP)-SVV, vestibular-evoked myogenic potential (VEMP), and posturography, did not show abnormalities in PPPD patients, high head-tilt perception gain (HTPG) in the HT-SVV test, an excessive perception of head tilt, appeared to be a specific marker for discriminating PPPD from other chronic vestibular diseases.

Postural sway was also examined in PPPD patients using wearable motion sensors [3]. Performance by PPPD patients in the sensory organization test (SOT) was poor; under low-demand conditions, patients showed greater anterior-posterior (AP) sway associated with visual dependence and medio-lateral sway than controls. These findings indicate the potential of AP sway as a diagnostic marker for PPPD.

Another study demonstrated that PPPD patients exposed to moving visual stimuli were more likely to develop gaze instability, which appeared to aggravate vestibular symptoms [4]. This phenomenon may provide insights into the neural mechanisms underlying the visual exacerbation of symptoms in PPPD patients. The lack of objective diagnostic tools motivated our hypothesis and study design.

Postural instability is associated with a diverse group of conditions, such as neurological diseases, vestibular, visual, and/or somatosensory dysfunctions, and musculoskeletal impairments. Posturography is a technique that is commonly employed to assess postural balance. It evaluates changes in the center of pressure (COP), defined as the center point of pressure exerted by the foot-ground contact surface on a force plate. Statokinesigrams, graphical representations of body sway measured by posturography, are commonly obtained and show the bidimensional trajectory of COP on the ground extracted by the COP plot. Parameters derived from COP trajectories to assess a subject’s capability and performance to maintain their balance generally include the sway path (cm) and sway area (cm^2^).

Visually induced dizziness is a symptom of PPPD that is exacerbated by visual stimuli. We previously showed that postural sway increased in PPPD patients exposed to several types of visual stimuli, indicating the potential diagnostic value of posturography with visual stimuli [5].

In the present study, we compared the validity of posturography with visual stimuli for the accurate diagnosis of chronic vestibular symptoms, including PPPD, central vestibulopathy, UVH, bilateral vestibular hypofunction (BVH), and PD.

## Materials and methods

The present study was approved by the Institutional Review Board of the National Hospital Organization Tokyo Medical Center (R20-161). All procedures involving human participants were performed in accordance with the ethical standards of the Institutional and/or National Research Committee and the 1964 Declaration of Helsinki.

Selection of patients

This study included six HCs, six patients with PPPD, three with PD, four with central vestibulopathy, 19 with UVH, and nine with BVH diagnosed at the Department of Otolaryngology - Head and Neck Surgery at National Hospital Organization Tokyo Medical Center.

All patients had vestibular symptoms for more than three months. Diagnoses were confirmed by various vestibular examinations, including posturography, the video head impulse test, caloric test, and cervical VEMP, which are described in detail below. Data collected on patients treated at the hospital between January 2021 and March 2022, and unselected consecutive patients with PPPD or UVH, were retrospectively analyzed. Hospital staff with no history of balance disorders and normal neurological function were recruited as subjects in the HCs group and were age- and sex-matched to the PPPD group.

Bárány Society diagnostic criteria for PPPD were used to diagnose patients in the present study [1]. Patients enrolled in the UVH group had vestibular symptoms for more than three months as well as abnormal unilateral values in the caloric test, video head impulse test, or cervical VEMP. Patients in the BVH group also had vestibular symptoms for more than three months and abnormal bilateral values in the caloric test, video head impulse test, or cervical VEMP. Patients in the central vestibulopathy group included those with vestibular migraine (N=3) [6] and dizziness after brain infarction (N=1).

Clinical symptom scales

The Dizziness Handicap Inventory (DHI)

DHI is a standard 25-item questionnaire that quantitatively assesses the impact of the symptoms of vestibular disorders on the daily life of patients [7,8]. The total score of DHI ranges between 0 (no disability) and 100 (severe disability).

The Niigata PPPD Questionnaire (NPQ)

NPQ [9] is a self-reported 12-item questionnaire that is used to screen PPPD patients and measure the extent of symptom exacerbation. It evaluates the degree of symptom exacerbation by an upright posture or walking, active or passive movement, and visual stimuli. Each factor is assessed using four questions, rated with scores of 0 (no symptoms) to 6 (intolerable). The maximum score for each factor is 24, and the total NPQ score is 72, with higher scores indicating more severe symptoms.

Vestibular function tests

Conventional posturography, the caloric test with electronystagmography, the video head impulse test, and the c-VEMP test were routinely performed on both sides for diagnostic purposes. If a peripheral vestibular disorder was not defined, brain imaging, including computed tomography (CT) and magnetic resonance imaging (MRI), was conducted.

Conventional posturography and posturography with various types of visual stimuli

Conventional posturography was the initial test used to diagnose patients. Static posturography was performed on a solid surface using Gravicoda R (ANIMA Corp., Tokyo, Japan) with eyes open and eyes closed. Posturography measured the area of sway with eyes open for 60 seconds. The same recording was then performed with eyes closed. The length of the path/s (LNG/TIME) and the environmental area (ENV) were employed as parameters for diagnosis. After conventional posturography, experimental posturography during visual stimuli was conducted with eyes open. The sampling rate was 20 Hz, and the filter was 6Hz.

**Figure 1:**
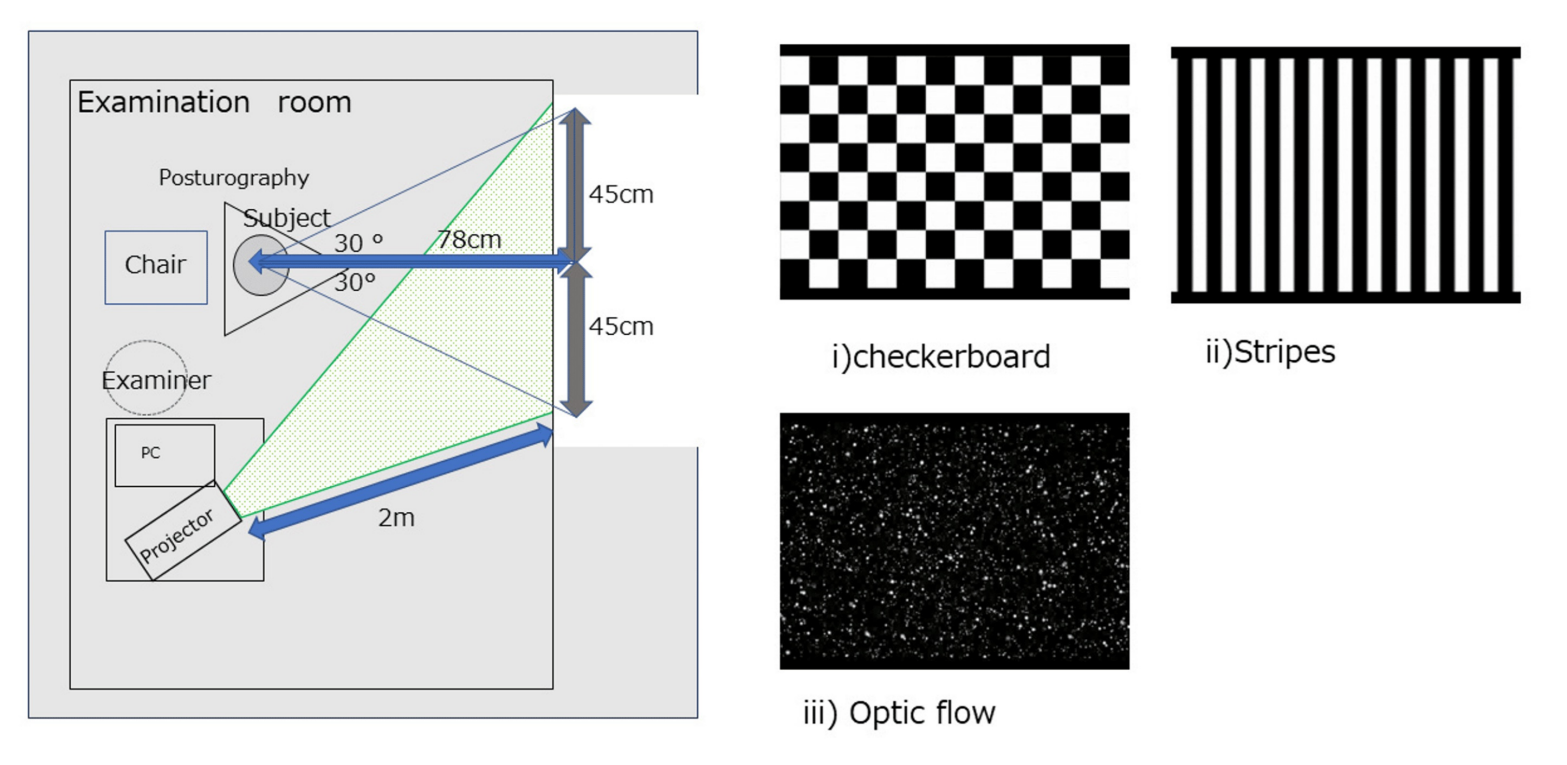
Setting and visual stimulation. Visual stimuli (checkerboard, vertical stripes, optic flow) were provided by Dr. Chihiro Yagi and have been previously described in Yagi et al., 2022 [4]. (i) Checkerboard pattern stimulus comprising 8 rows × 12 columns of squares reversed in contrast (100%) at 12 Hz. (ii) Optokinetic stimulus with 12 black-and-white vertical stripes sweeping across a screen at 6 seconds. (iii) Radial optic flow stimulus with moving white dots (size: 0.1-1.1 degrees of visual angle, speed: 3 seconds with a flat speed gradient) on a black background expanding from the center of the screen.

The visual stimuli were adapted from prior protocols validated to provoke visually induced dizziness in similar patient populations [10]. Visual stimuli in daily life that may aggravate PPPD symptoms include (i) ﬂashing lights on a television, (ii) scenery moving sideways when viewed from the inside of a train, and (iii) scenery ﬂowing from front to back when sitting in the passenger seat of a car; therefore, the three types of moving visual stimuli used for recordings in the gaze stability test were created in an attempt to reproduce these stimuli. Movies with moving visual stimuli, described in a previous study [4], were provided by Dr. Chihiro Yagi. Examination settings are shown in Figure 1.

The visual angle of the screen was 60 degrees. Figure 1 shows the order of stimuli in the gaze stability test. The following moving visual stimuli were presented on a projector screen continuously for 60 secs each: (i) a checkerboard pattern stimulus of 8 rows × 12 columns of squares reversed in contrast (100%) at 12 Hz, (ii) an optokinetic stimulus of 12 black-and-white vertical stripes sweeping across the projector screen at 6 seconds, and (iii) a radial optic ﬂow stimulus of moving white dots (size: 0.1-1.1 degrees of the visual angle, speed: 3 seconds with a ﬂat speed gradient) on a black background expanding from the center of the screen. The comparison between before and during visual simulation was conducted. The ratios of LNG and ENV before and during the visual stimuli were calculated as the visual ratio (VR) with the eye open by dividing post-stimuli values for LNG and ENV by their pre-stimuli values (post-LNG and ENV/pre-LNG and ENV).

Statistical analysis

Statistical analyses were performed using GraphPad Prism version 8 (GraphPad Software, San Diego, CA, USA). The Kruskal-Wallis test was used for comparisons, and a p-value of <0.05 was considered statistically significant. Data are presented as mean ± standard deviation (SD) unless otherwise indicated. Group comparisons were conducted using the Kruskal-Wallis test, followed by Dunn’s post hoc test for multiple comparisons to identify significant differences between groups.

## Results

Subject characteristics

Subject characteristics are summarized in Tables 1-2. No significant differences were observed in age or in the scores of the DHI or NPQ between the groups.

**Table 1:** Subject characteristics. Values are shown as mean ± standard deviation (SD) unless otherwise indicated. PD: psychogenic dizziness; PPPD: persistent postural-perceptual dizziness; UVH: unilateral vestibulopathy; BVH: bilateral vestibulopathy; HCs: healthy control; N: number of subjects; DHI: Dizziness Handicap Inventory; DHI-P: Physical Subscale (of DHI); DHI-E: Emotional Subscale (of DHI); DHI-F: Functional Subscale (of DHI)

	N	Male	Female	Age	DHI-P	DHI-E	DHI-F	DHI total
Central	4	4	0	51.5±23.9	15.5±1.9	17±7.5	17.5±8.6	50±14.8
PD	3	2	1	35.3±10.2	6±2	16±8.7	13.3±4.6	35.3±13.3
PPPD	6	2	4	51.8±10.6	17±3.2	16±7.1	14.6±9.0	47.6±17.4
UVH	19	7	12	59.8±13.6	8.1±7.4	10.2±11.1	9.3±11.3	27.6±27.8
BVH	9	2	7	57.6±23.0	8.8±9.9	10.7±14.1	8±11.1	27.6±31.1
HCs	6	5	1	37.4±10.4	NA	NA	NA	NA

**Table 2:** Subject characteristics. Values are shown as mean ± standard deviation (SD) unless otherwise indicated. PD: psychogenic dizziness; PPPD: persistent postural-perceptual dizziness; UVH: unilateral vestibulopathy; BVH: bilateral vestibulopathy; HCs: healthy control; NPQ: Niigata PPPD Questionnaire; NPQ-U: upright posture/walking factor of NPQ; NPQ-M: movement/active motion factor of NPQ; NPQ-V: visual stimulation factor of NPQ; A: anxiety; D: depression

	NPQ-U	NPQ-M	NPQ-V	NPQ total
Central	13±6.6	16±4.0	14.7±2.9	43.7±7.9
PD	8.6±13.3	8±1.7	10±1.7	26.6±5.5
PPPD	11.3±3.9	14±2.7	13.8±5.1	39.1±10.3
UVH	4.1±4.9	5.7±5.9	5.8±7.2	15.7±17.6
BVH	7±8.7	8.1±7.6	4.8±7.2	20±22.0
HCs	NA	NA	NA	NA

Pre- and post-stimulation posturography measures

**Figure 2:**
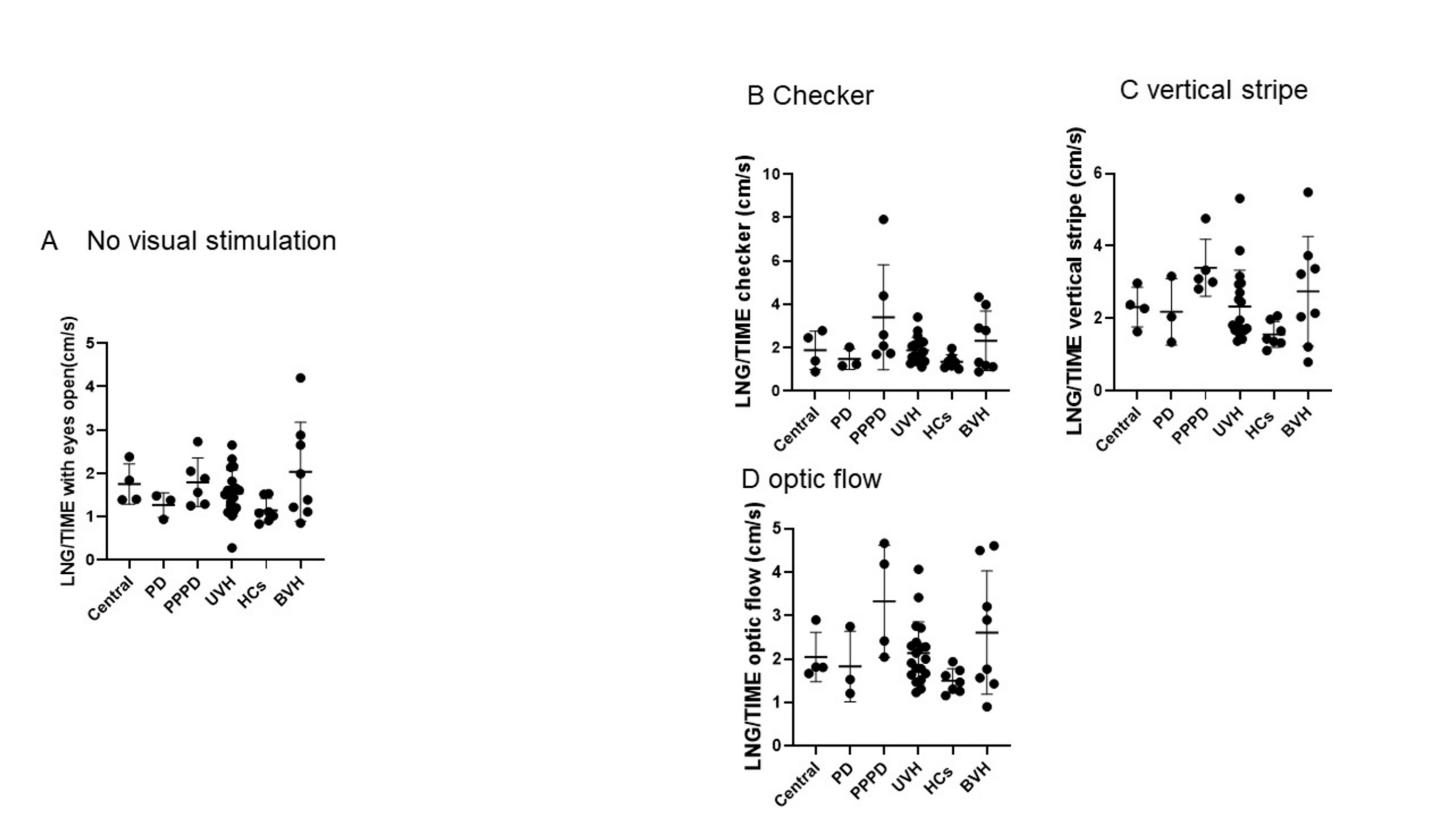
LNG/TIME with and without visual stimulation. The value of sway path length per unit time (LNG/TIME) did not show a statistically significant difference under any of the four different conditions (A: no visual stimulation, B: checkerboard pattern, C: vertical stripe pattern, and D: optic flow). Each bar in the figure represents the median value for the corresponding group. PD: psychogenic dizziness; PPPD: persistent postural-perceptual dizziness; UVH: unilateral vestibular hypofunction; BVH: bilateral vestibular hypofunction; HC: healthy controls

**Figure 3:**
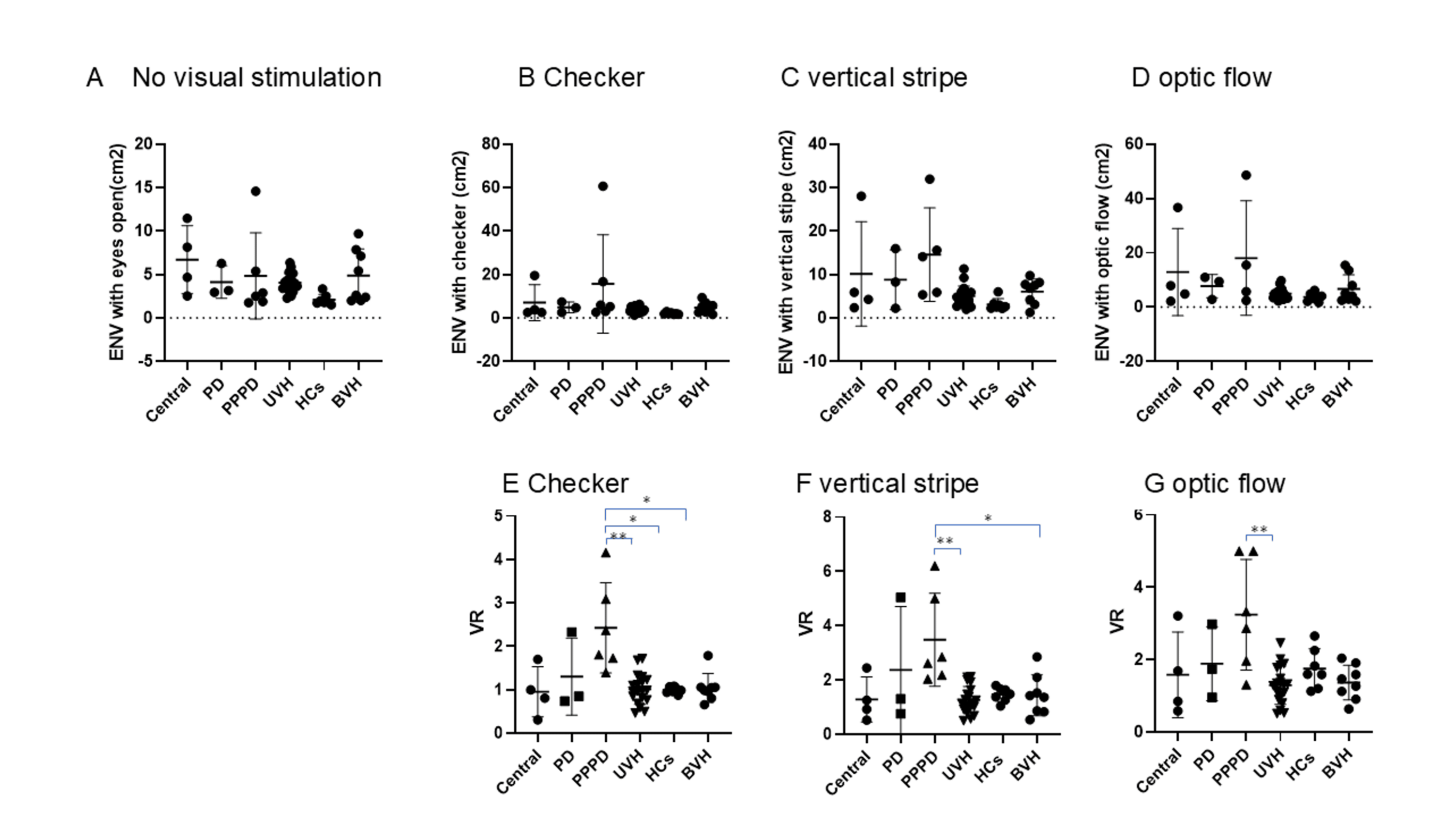
ENV and VR with and without visual stimulation. The value of envelope area (ENV) did not show a statistically significant difference under any of the four conditions (A: no visual stimulation, B: checkerboard pattern, C: vertical stripe pattern, and D: optic flow). However, the visual ratio (VR) of patients with persistent postural-perceptual dizziness (PPPD) showed a statistically significant difference under all three visual stimulation conditions (E: checkerboard, F: vertical stripes, and G: optic flow). See p-values in Table 3. Each bar in the figure represents the median value for the corresponding group. PD: psychogenic dizziness; UVH: unilateral vestibular hypofunction; BVH: bilateral vestibular hypofunction; HC: healthy controls

There were no significant group differences in LNG/TIME or ENV values either before or after visual stimulation (Figures 2-3). Each bar in the figures represents the median value for the corresponding group.

VR under stimulation

The VR for both LNG/TIME and ENV was larger in the PPPD group compared to other groups (Figure 3). Statistical analysis was conducted using the VR of ENV under each visual stimulus condition:

Checkerboard Stimulation

VR was significantly larger in the PPPD group than in the central vestibulopathy, UVH, BVH, and HCs groups (p < 0.05), but not significantly different from the PD group (Table 3).

**Table 3:** Adjusted p-values for comparisons of VR between PPPD and subjects with various vestibular disorders. VR: visual ratio; PPPD: persistent postural-perceptual dizziness; UVH: unilateral vestibulopathy; BVH: bilateral vestibulopathy; HCs: healthy control * p < 0.05, ** p < 0.01

VR of PPPD	Checkerboard	Vertical stripes	Optic flow
Central	0.0254	0.0307	0.1829
Psychogenic	0.1162	0.3485	>0.9999
UVH	0.0033**	0.0018**	0.0079**
BVH	0.0181*	0.0354*	0.0686
HCs	0.0276*	0.1657	0.753

Vertical Stripes Stimulation

VR was significantly larger in the PPPD group than in the central, UVH, and BVH groups, but not significantly different from the PD or HC groups (Table 3).

Optic Flow Stimulation

VR was significantly larger in the PPPD group than in the central and UVH groups, but not significantly different from the PD, BVH, or HC groups (Table 3).

## Discussion

In the present study, posturography was performed before and during various visual stimuli in an attempt to discriminate PPPD from other chronic vestibular diseases. Recently, with respect to functional dizziness, the spectral content of posturography is one of the topics of research. We dared to focus not on the spectrum but on the basic parameters at this time. It would be interesting to see the spectrum content in the later study. The results obtained revealed that VR in the PPPD group was significantly different from that in the other groups tested. Among the three different visual stimuli employed - the checkerboard, stripes, and optic flow - the checkerboard stimulus was the most robust for discriminating PPPD from the other diseases. However, no significant differences were observed between the PPPD and PD groups. Conducting further subgroup analyses and including larger samples could help to clarify these overlaps, which is essential for improving diagnostic accuracy. The PD group included only three patients with a wide range of psychiatric disorders, such as depression and anxiety disorders, including panic disorder. Therefore, the SD of the PD was very large, which resulted in difficulties discriminating between these two groups.

Posturography is performed to assess postural balance both objectively and quantitatively. It has been proposed as a tool to assist in the differential diagnosis of balance disorders and is employed in evaluations of the severity of postural imbalance as well as the impact of therapeutic interventions [10,11].

In patients suffering from chronic or functional dizziness, studies have shown that posturography with or without the addition of visual conflict stimuli can enhance diagnostic accuracy. For instance, Dieterich and Staab reported that instrumented posturography incorporating visual stimuli improved the identification of syndromes resembling PPPD [11]. Likewise, Wrisley et al. found that the SOT, a specific form of posturography, was effective in quantifying postural sway variability when compared to standard vestibular tests or healthy controls [12]. These findings suggest that posturography contributes to more accurate differentiation among subtypes of balance disorders.

In the present study, we used various visual stimuli to induce dizziness in PPPD patients. Visually induced dizziness is a hallmark of PPPD [1], and our findings are consistent with this characteristic. Although the recommended use of SOT is to assess impairments in postural stability, not to diagnose patients, it may be applied to identify patients, such as those with PPPD, with impairments in postural control and sensory integration that differ from those with peripheral or central vestibulopathies. The PPPD group developed postural instability during posturography with various types of visual stimuli. Deficits in visual, somatosensory, and multimodality integration were observed in this group; however, some patients performed poorly, whereas others had less difficulty completing the test. Computerized dynamic posturography has the potential for identifying specific patterns of functional impairment in PPPD patients that are consistent with emerging findings on the brain mechanisms underlying PPPD.

Previous studies attempted to discriminate PPPD patients using clinical examinations. Vestibular symptoms in PPPD are induced by visual stimuli and body movements, suggesting sensory hypersensitivity in the sensory systems involved in maintaining postural stability. The HT-SVV test, which measures SVV with the head tilted, is more sensitive than the SVV test. HTPG calculated from SVV at head tilt and the actual head tilt angle (head tilt angle) were significantly greater in PPPD than in uncompensated unilateral vestibular dysfunction or psychogenic vertigo. With HTPG > 1.202, the specificity of the PPPD diagnosis was 95.2%. Since UP-SVV without head tilt and VEMP, a gravity-sensing otolithic system brain test, were normal, deep neck hyperalgesia due to head tilt is present in PPPD patients, and HTPG is expected to become an objective index for its diagnosis [2].

PPPD is characterized by visually induced dizziness, which persists after the end of the visual stimulation. When the different groups were asked to fixate on a single point before and after a visual stimulation to induce vertigo in the present study, the PPPD group had a large eye sway, even one minute after the end of the visual stimulation, indicating that their ability to fixate on a single point was impaired. Therefore, the mechanism underlying dizziness that persists after a visual stimulation is a reduction in the fixation function. The fixation function test before and after a visual stimulation has potential for the specific detection of PPPD [4].

Regarding its pathogenesis, PPPD is a functional disease similar to irritable bowel syndrome in gastrointestinal disorders; however, it may be complicated by psychiatric or organic diseases. In other words, some organic conditions, such as acute vestibular disease, may precede PPPD and are modified by psychological factors, including anxiety, which may be the primary or a secondary condition, leading to the development of PPPD as a functional disorder [1]. This is similar to the process of bacterial enteritis as an organic disease, which transitions to irritable bowel syndrome as a functional disease.

Indovina et al. [10] summarized the functional brain imaging findings of PPPD reported to date and found that local activity and functional connectivity in the vestibular sensory cortex were reduced, while local activity and functional connectivity in the visual cortex were enhanced. Therefore, PPPD reduces local activity and functional connectivity in the vestibular sensory cortex, but enhances functional connectivity in the visual cortex, with the hippocampus and precuneus being involved in spatial cognition, the prefrontal cortex controlling attention and emotional responses, and the motor cortex controlling posture. These findings suggest that visual input is more important than vestibular input and that changes in visual-motor and visual-emotional network interactions may be factors contributing to the pathophysiology of PPPD. The present results provide insights into this phenomenon.

There are several limitations that should be acknowledged. First, the sample size was too small to establish a definitive cut-off value for diagnostic purposes. Second, posturography with visual stimuli can sometimes provoke significant postural instability, posing a risk of falls; therefore, the presence of at least two examiners is required to ensure patient safety. Third, the visual stimulation setup necessitates a large screen and sufficient physical space, which may limit its feasibility in smaller clinical settings. Lastly, due to the large SD observed in the PD group, it was difficult to clearly differentiate PPPD from PD based on posturographic parameters. These limitations should be addressed in future studies.

## Conclusions

Exposure to moving visual stimuli, particularly checkerboard patterns, induced greater postural instability in patients with PPPD compared to other vestibular conditions. This suggests that visual-stimulus posturography may serve as a useful diagnostic aid for PPPD in clinical practice. Furthermore, these findings contribute to a deeper understanding of the neural mechanisms by which visual stimuli exacerbate symptoms in PPPD patients.
